# Enhanced recovery care after colorectal surgery in elderly patients. Compliance and outcomes of a multicenter study from the Spanish working group on ERAS

**DOI:** 10.1007/s00384-016-2621-7

**Published:** 2016-07-04

**Authors:** Santiago Gonzalez-Ayora, Carlos Pastor, Hector Guadalajara, Jose Manuel Ramirez, Pablo Royo, Elizabeth Redondo, Antonio Arroyo, Pedro Moya, Damian Garcia-Olmo

**Affiliations:** 1 Department of General Surgery, Division of Colorectal Surgery, Fundacion Jimenez-Diaz, Reyes Catolicos Ave #2, 28040 Madrid, Spain; 2 Department of General Surgery, Division of Colorectal Surgery, Hospital Clinico Universitario, Zaragoza, Spain; 3 Department of General Surgery, Division of Colorectal Surgery, Hospital General Universitario, Elche, Spain

**Keywords:** ERAS, Enhanced, Colorectal cancer, Elderly

## Abstract

**Purpose:**

ERAS (enhanced recovery after surgery) programs have proven to reduce morbidity and hospital stay in colorectal surgery. However, the feasibility of these programs in elderly patients has been questioned. The aim of this study is to assess the implementation and outcomes of an ERAS program for colorectal cancer in elderly patients.

**Methods:**

This is a multicenter observational study of a cohort of elderly patients undergoing colorectal surgery within an ERAS program. A total of 188 consecutive patients over 70 years who underwent elective colorectal surgery within an ERAS program at three institutions during a 2-year period were included. The compliance with the ERAS protocol interventions was measure. Complications were evaluated according to Clavien-Dindo classification. Data on length of stay and readmission rates were analyzed.

**Results:**

Early intake and early mobilization were the most successfully carried out interventions. There was a global compliance rate of 56 % of patients for whom compliance was achieved with all measured interventions. The median hospital length of stay was 6 days. Almost 60 % of patients had no complications, 24 % had minor complications while 13 % had major complications; of them, 8 % patients were reoperated. The readmission rate was 6.4 %.

**Conclusions:**

ERAS after colorectal surgery in elderly patients presents as safe and feasible based on good reported outcomes of compliance rates, complications, readmissions, and needs for reoperation.

## Introduction

Over the last decade, the enhanced recovery after surgery (ERAS) programs have generated a true revolution in colorectal surgery. This revolution has been compared to the innovative concept of total mesorectal excision for rectal surgery [1] or to that of the adoption of laparoscopic surgery as the gold standard in colon surgery [2, 3].

**Fig. 1:**
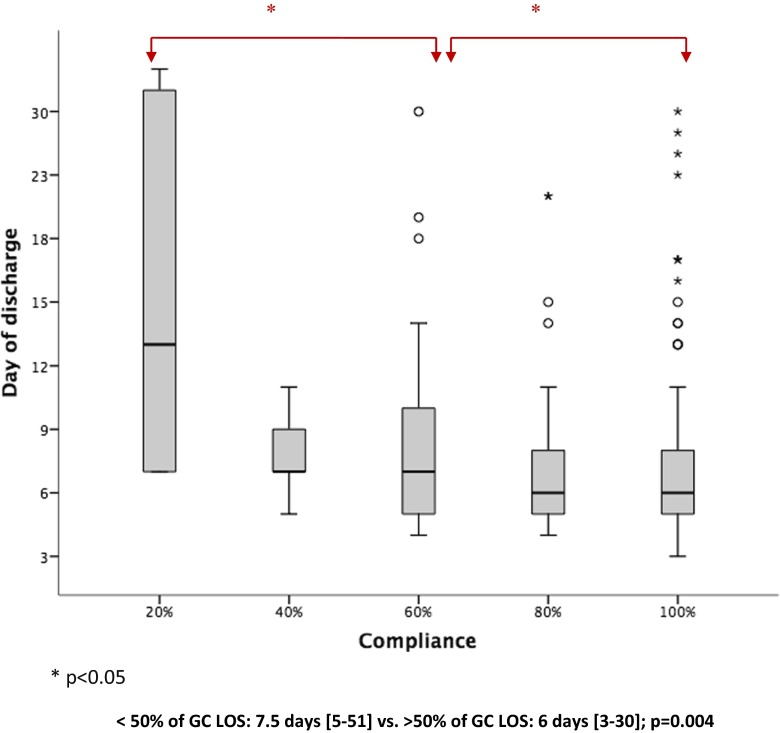
Influence of Compliance with ERAS measures in LOS (Kruskal-Wallis test)

**Fig. 2:**
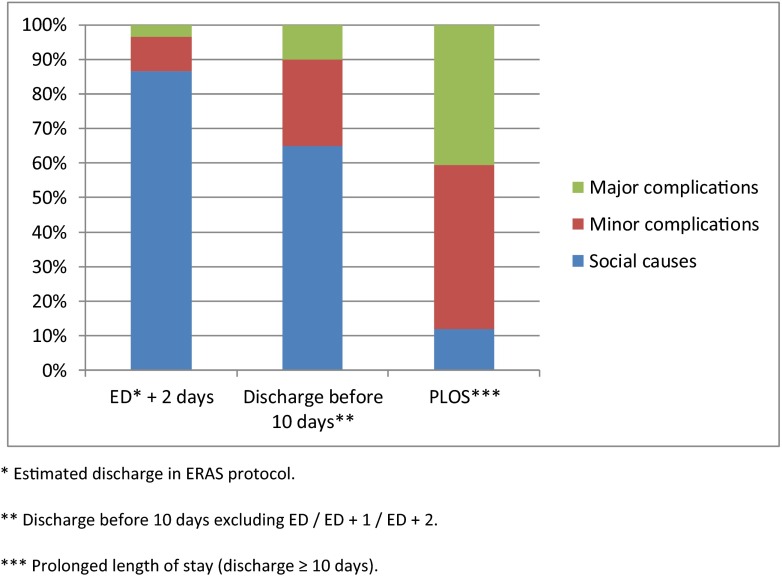
Reasons for delaying discharge beyond ERAS estimated date. *Estimated discharge in ERAS protocol

Initially proposed by Professor Henrik Khelet from Denmark, ERAS is a multidisciplinary set of care interventions in patients with the goal to obtain a comprehensive recovery after any surgical intervention [4, 5]. Focused on colorectal cancer surgery, there exist to date strong evidence demonstrating that adhesion to ERAS protocols can minimize morbidity by decreasing secondary complications while also being cost-effective in shortening the length of hospital stay (LOS) [6–11]. Along with all the benefits of ERAS, it is known that there are significant limitations in implementing these protocols, due to the fact that compliance of all interventions may be difficult to achieve by each patient, which may worsen end results [12, 13]. Therefore, most studies have excluded by definition elderly patients from ERAS pathways. The reason for such exclusion was that adherence to protocols in elderly patients was assumed to be unfeasible due to physical impairments or associated comorbidities [14]. However, currently over 70 % of colorectal cancers are primarily diagnosed among patients over the age of 65 [15], and it seems reasonable that ERAS should be targeting elderly patients with associated comorbidities rather than young, healthy patients. A recent systematic review from the UK concludes that there currently exists a lack of evidence regarding ERAS in elderly patients and further studies are required to assess the implementation grade and possible benefits of ERAS protocols in this particular population [13].

This led to the design of the present multicenter study to test the hypothesis of whether it is feasible to implement an ERAS program in elderly patients undergoing elective, colorectal surgery by assessing compliance with interventions and postoperative outcomes.

## Materials and methods

A retrospective analysis was performed from the GERM Group (Spanish for Grupo Español de Rehabilitacion Multimodal) prospective database by selecting patients ≥70 years, undergoing colon and rectal surgery following ERAS protocols in the last 2 years (2013–2014). Data was obtained from three university tertiary centers in Spain, Hospital Universitario Fundacion Jimenez-Diaz (Madrid), Hospital Clinico Universitario (Zaragoza), and Hospital General Universitario (Elche), of consecutive patients with elective colon and rectal resections excluding urgent and palliative surgeries. A multidisciplinary task force composed of surgeons, anesthesiologist, nurses, and physical therapists was convened in each center at the beginning of the study to implement and monitor compliance with interventions. A nurse coordinator was assigned to each center to routinely assess ERAS compliance obtained from the patient’s medical records and to report monthly the results to the study committee. In addition, a videoconference meeting was held every 3 months involving all three centers. The study was initiated after obtaining approval by the local institutional review board committee from each institution.

A bundle of 10 interventions were adopted at the same time in all three participating hospitals on the basis of our previously published protocol [16, 17] including the following: (1) preoperative advice and evaluation of nutritional status, (2) intravenous iron supplementation in cases of preoperative anemia, (3) avoidance of full mechanical preparation for colon resections, (4) administration of carbohydrate-rich drinks 1 day prior and on the morning of surgery, (5) goal-directed intraoperative fluid therapy and body temperature control during surgery, (6) the use of intraoperative pneumatic legs compression, (7) avoidance of nasogastric tubes and drains when possible, (8) taking in oral fluids during the early postoperative period (means 6–8 h after surgery) and soft-food diet by the second postoperative day and early mobilization (walking from the bed to the sofa at 6–8 h after surgery), (9) intravenous fluid restriction and removal of urinary catheter (indicates stopping fluids and catheter removal by the first postoperative day), and (10) multimodal analgesia (epidural catheter for open surgery cases).

By definition, we targeted hospital discharge date at fourth or fifth postoperative day for colon or rectal surgery, respectively. Patients could be discharged if they met the following criteria: good mobilization, adequate oral intake for liquids and solids, recovered gastrointestinal transit minimally for passing gas, normal urinary outputs, no wound problems, good pain control with oral medication, absence of fever in the last 48 h, C-reactive protein below 10 mg/L at discharge, and showing a decreasing trend in previous laboratory test. In addition, the patient and family needed to feel comfortable with the discharge and information given regarding possible complications and early detection.

Compliance with all interventions was combined and expressed as the percentage of patients who had correct intervention and documentation. Global compliance (GC) was defined as the rate of patients for whom compliance was achieved with all measures of the ERAS protocol.

The POSSUM score (Physiological and Operative Severity Score for the enumeration of Mortality and Morbidity) [18] was determined to calculate postoperative morbidity and mortality risks. Additional operative variables such as a stoma construction and the use of a laparoscopic surgical approach were also recorded. Short-term postoperative complications were graded using the Clavien-Dindo classification [15]. LOS and rates and causes of readmission during the first 30-day postoperative period were also documented. A further analysis was performed when LOS was prolonged to identify the causes of delayed hospital discharge.

### Statistical analysis

Patient baseline characteristics at the time of surgery, age, gender, American Society of Anesthesiologists (ASA) score, major comorbidities, preoperative anticoagulation therapy were obtained from each patient’s electronic medical record. Compliance information was gathered from inpatient medical charts and recorded on a monthly basis in the database. Any measure during the process was considered as “non-compliant” if documentation was incomplete. Descriptive statistics are presented using *t* test with mean and standard deviation (SD) or median and range for continuous variables. Comparison of differences between group means was carried out using ANOVA for variables with normal distribution, and the Mann-Whitney test for continuous variables with non-parametric distribution. We used chi-squared analysis with Fisher’s exact test when any value observed in the contingency table was less than 5 to compare proportion variables. We performed a Kruskal-Wallis test to explore the impact of compliance within interventions on the length of stay. The level of statistical significance was taken as *p* < 0.05. All statistical calculations were performed using SPSS software® (version 22.0, SPSS Inc., Chicago, IL).

## Results

Throughout the study period a total of 188 patients were treated among the three centers. Of them, 109 (58 %) were men, 79 (42 %) were women with a median age for the whole group of 79 [70–93] years old. Demographics, patient baseline characteristics, and surgical procedures are presented in Table 1. It is important to note that sigmoid resection and right and left colectomies were the most common surgery; 77 % (*n* = 145) performing a primary anastomosis. We also included rectal cancer surgeries (*n* = 43, 23 %); of them, 34 patients in whom low anterior resections were done with or without a temporary stoma. By summarizing these cases, 95 % of patients had a primary anastomosis with sphincter preservation. In addition, almost 45 % of surgeries (40 % colectomies; 58 % rectal surgeries) were performed via laparoscopic approach.

**Table 1:** Patient baseline characteristics and surgical techniques

Variable	*n* = 188	
Age (years)^a^	79	[70–93]
Male	109	58 %
Female	79	42 %
POSSUM score (morbidity)^a^	29.3 %	[6.9–80.5]
POSSUM score (mortality)^a^	5.3 %	[1.3–26.8]
Comorbidities		
Diabetes	52	28 %
Anticoagulant treatments	36	19 %
Surgical technique		
Colon surgery	145	77.1 %
Laparoscopic colon surgery	58	40.0 %
Rectal surgery	43	22.9 %
Laparoscopic rectal surgery	25	58.1 %
Total laparoscopic surgery	83	44.1 %
Surgical procedure		
Right colectomy	80	42.6 %
Left colectomy	15	8.0 %
Sigmoidectomy	50	26.6 %
Low anterior resection	34	18.1 %
Hartmann	7	3.7 %
Abdominoperineal resections	2	1.1 %
Stoma (temporary or definitive)	25	13.3 %

^a^Median [range]

The POSSUM scoring system was calculated for each patient immediately after the surgery, with a median of 29.3 % [6.9–80.5] and 5.3 % [1.3–26.8] for expected postoperative morbidity and mortality, respectively.

### Compliance outcomes

Data was measured independently for each ERAS intervention as is presented in Table 2. By definition, we avoided mechanical preparation in colonic surgeries, and all patients received preoperative dietary recommendations and a carbohydrate-rich drink at 2–4 h before surgery. Early intake of clear liquids at 6 h after surgery and early mobilization were the most successfully carried out interventions in over 90 % of patients. On the other hand, the discontinuing early of intravenous fluids and early removal of urinary catheter rates were 73 and 64 %, respectively.

**Table 2:** Compliance rates with ERAS interventions

Variable		*n* (%)
No drainage^a^		81 (43.0)
Epidural anesthesia^a^		116 (61.7)
Early intake		173 (92.0)
Early suspension of intravenous fluids		138 (73.4)
Early mobilization		169 (89.9)
Early urinary catheter removal		122 (64.9)
Global compliance		105 (56.0)
Variable	Global compliance (%)	*X* ^2^ *p* value
Laparoscopic surgery Open surgery	59.0 53.3	0.43
Colon surgery Rectal surgery	60.0 41.9	0.03
Without stoma^b^ With stoma^b^	58.9 36.0	0.03

^a^ERAS interventions not included in the global compliance

^b^Temporary or definitive stoma

Overall, there was a GC rate of 56 % of patients for whom compliance was achieved with all measured interventions. When performing laparoscopic surgery a higher, but non-significant GC percentage was achieved (59 % lap vs. 53.3 % open; *p* = 0.43). Patients who underwent rectal surgery had significantly lower GC rates when compared to colon surgery (41.9 % rectal vs. 60.0 % colon; *p* = 0.03). In addition, a construction of either a temporary or a definitive stoma, also decreased GC rates (36.0 % with stoma vs. 58.9 % without stoma; *p* = 0.03).

### Postoperative outcomes

Postoperative outcomes, surgical complications, and length of stay are showed in Table 3. About 60 % of patients had an uneventful postoperative course without complications (66.7 % when GC achieved vs. 56.6 % without GC), 25 % had minor complications, prolonged ileus being the most common complication. Important to note, we did not observe frequent medical complications in elderly patients such as postoperative pneumonia or venous thromboembolism. Major complications occurred in 13 % of cases, including 15 patients who had a clinical anastomotic leakage (8 %). The need for a reoperation was 8.5 % (16 patients) mainly due to anastomotic leakage. Mortality following complications occurred in three patients (1.6 %). As expected, patients who had rectal surgeries suffered worse complications compared to patients with colon surgeries and with a higher risk of anastomotic leakage.

**Table 3:** Postoperative outcomes

Postoperative complications	*n* (%)	*X* ^2^ *p* value
No complications	117 (62.2)	
Dindo-Clavien grades I–II	46 (24.5) with GC 30 without GC 20	0.32
Dindo-Clavien grades III–V	25 (13.3) with GC 13.4 without GC 13.2	0.34
Postoperative ileus	44 (23.4) with GC 21.0 without GC 26.5	0.37
Anastomotic leakage	15 (8.0) with GC 7.6 without GC 8.4	0.83
Mortality	3 (1.6)	
Reoperations Anastomotic leakage Bowel ischemia Hemoperitoneum Internal hernia Urinary tract injury	16 (8.5) 10 (5.3) 2 (1.0) 1 (0.5) 1 (0.5) 1 (0.5)	
Hospital length of stay^a^	6.0 [3–51] with GC 6 [3–30] without GC 7 [4–51]	0.03
Readmissions Abdominal abscess Wound infection Late anastomotic leakage Dehydration	12 (6.4) 3 (1.6) 3 (1.6) 2 (1.0) 3(1.0)	

^a^Median [range]

When investigating the impact of GC on postoperative complications, we observed the following data as shown in Table 3: (1) a clinical, but not statistically significant 10 % reduction on minor Clavien-Dindo I/II complications (30 % with GC vs. 20 % without GC, *p* = 0.32). While we did not observed influences of achieving GC on major Clavien-Dindo III/IV complications (13.4 % with GC vs. 13.2 % without, *p* = 0.34), (2), a lower percentage of postoperative ileus (21 % with GC vs. 26.5 % without GC; *p* = 0.37), and (3) a non-significant effect of GC in terms of anastomotic leakage was present (7.6 % with GC vs. 8.4 % without GC; *p* = 0.83).

Median LOS was 6 [3–51] days for the entire study population including the readmission days when occurred. A significant impact of GC on LOS was observed with a median reduction of 1 day in LOS including readmissions (6 [3–30] days with GC vs. 7 [4–51] days without GC; *p* = 0.03).Figure 1 shows the influence of percentage of GC in reduction of median LOS. A significant reduction of 1.5 days in LOS was achieved when we were able to reach a 50 % rate in GC (7.5 [5–51] days < 50 %GC vs. 6 days [3–30] days > 50 %;*p* < 0.04]Figure 2 shows further analysis of LOS based on the postoperative day, showing that 41 % of the study population were discharged at ERAS or ERAS + 1 day periods. The main reasons for a delayed discharge are shown in Fig. 2. indicating social issues to be the most observed reasons for delay in discharge compared to postoperative complications. Readmission to the hospital occurred in 12 patients (6.4 %). The most common cause of readmission was deep and organ/space (intra-abdominal) infections requiring CT-guided percutaneous drainage and/or antibiotic coverage. There were two cases of late anastomotic leakage with pelvic abscesses in patients who underwent low anterior resections

## Discussion

Our data shows reliable outcomes from a multicenter, observational, non-randomized study group in the implementation of a standardized ERAS protocol in elective colorectal surgery in elderly patients.

There is a lack of information on the exact impact of ERAS interventions in elderly patients, although the current evidenced-based data has been recently reviewed in a systematic review from the UK [13]. This review reported to date two clinical trials comparing ERAS with non-ERAS, focused on elderly patients showing in favor of ERAS, a shorter length of stay and a significant decrease in minor complications [19, 20]. However, one of these studies had a low number of patients, did not show data about rates of reoperation or readmission, and did not report compliance with interventions. Due to lacking such data, our study proved that a sustained effort from a dedicated multidisciplinary team could achieve a high level of compliance rates with most ERAS interventions in both colon and rectal surgeries focused in elderly patients.

When analyzing compliance with ERAS bundles, we obtained the goal of 90 % compliance within two interventions: early intake of clear liquids and early mobilization when planned. Both were difficult to be implemented and required an extra effort from the caregivers, as most elderly patients were reluctant to get out of bed or drink liquids 6–8 h after surgery. In our experience, management of proper pain control within over 60 % of patients with epidural morphine-sparing analgesia can help to accomplish early mobility from the bed to the sofa without adding secondary effects. Moreover, almost half of the participants where operated via laparoscopic approach, adding advantages in achieving these compliance rates. From the rest of ERAS interventions, early stopping of intravenous fluids and early urinary catheter removal showed the lowest compliance rates (73 and 65 %, respectively). We believe the reason for this delay was due to short urinary outputs in elderly patients during the first postoperative day leading to a delayed removal of the urinary catheter. Instead of looking at each specific intervention, we developed the variable global compliance, defined as the rate of patients for whom compliance was achieved with all the measurements of the ERAS protocol. Overall, there was a GC rate of 56 % in the study population. Identified barriers to achieve a higher GC with statistical differences were rectal surgeries, the creation of a stoma and open surgery cases. Therefore, a great effort should be made to increase our plan of care in patients with these characteristics.

Criticism of ERAS protocols will argue that a high readmission rate especially in elderly patients will invalidate any positive result. Regarding 30-day postoperative outcomes, our data showed that 62 % of patients had no complications, 25 % had minor complications, and 13 % suffered major Clavien-Dindo’s complications. Postoperative ileus was the most common observed complication in almost 25 % of patients who required a nasogastric tube and prolonged total parenteral nutrition. We did not observe respiratory complications such as pneumonia or pulmonary edema or cases of deep vein thrombosis. Reoperation was needed in 8.5 % of patients and clinical anastomotic leakage occurred in 8 % for colon surgeries and 11.6 % in rectal surgeries.

An impact of GC on decreasing postoperative complications has been earlier reported in 2011 by a multicenter study from the European ERAS Study Group especially when GC could be achieved by over 70 % [21]. When analyzing the impact of GC in postoperative complications, there was a clinical, but not significant reduction of 10 % in minor complications and about 5–6 % in postoperative ileus, while mayor postoperative complications such as anastomotic leakage remained unchanged. Our aim was to establish and detect an impact of GC in decreasing complications; however, a small sample size may have underpowered the effect of GC in complications in our study.

For predicting patients at risk of developing complications, we assessed for each patient their POSSUM score. We believed that the POSSUM score might be more useful than the ASA score to predict postoperative outcomes and that it is adequate to assess patient’s baseline performance status; however, it is not a valid tool to identify patients at risk of failure in the ERAS programs. The key point would be to select in the preoperative evaluation who will fail in achieving ERAS, to either design a personalized program for those patients, or have the opportunity to improve their conditions in the prehabilitation period. In this sense, a better score taking into account “frailty” rather than “elderly” using the modified frailty index has demonstrated to correlate better with complications, longer lengths of stay, and readmissions and has recently been validated in elderly patients undergoing colorectal surgery under ERAS protocols [22]. Therefore, we would recommended before implementing ERAS in elderly patients to use a prospective score to identify patients at risk for not achieving the protocol so resources and postoperative supports would be better allocated.

Contemporary postoperative admission stays in ERAS protocols range from 3 to 5 days [23] in comparison to traditional practice of up to 5 to 9 days. Focused on surgery in elderly patients, a prospective study of 87 patients >70 years old, reported a mean LOS of 3.9 days [24]. LOS is often used as a surrogate marker of recovery, and it should not be offset by a higher rate of hospital readmission.

In our data, the median LOS was 6 days for the entire study population with minimal differences between hospitals. When analyzing LOS by subgroups, 41 % of patients were discharge at the estimated day in ERAS protocols (in the fourth postoperative day for colon surgery or fifth postoperative day for rectal surgery) or ERAS + 1 day. Moreover, when analyzing the reasons for a delayed discharge, a needed of social support and non-postoperative complications were the most common causes for patients to be discharged from the hospital before the 10th postoperative day. We believe this to be because two of the hospitals from the study are reference hospitals covering suburban and rural areas. These patients may live a greater distance from the hospital, making physicians reluctant to discharge them earlier. Important to note, our data showed a positive effect of GC in LOS when we were able to achieve, at least, 50 % of compliance with the interventions.

Readmission to the hospital after discharge was observed in 6.4 % of patients, mainly due to abdominal abscess after pelvic surgeries. We reported two cases of late anastomotic leakage in rectal cancer surgeries in patients who were readmitted to the hospital needing percutaneous drainage. In contrast, there were no cases of delayed leakage in colon resections. We believe that, based on the study population of elderly patients, these LOS and readmissions rates are considerably good and support the idea that ERAS is a feasible and secure option for this particular population.

Our study has some limitations that deserve to be mentioned. First, although this was a multicenter study, it was not being conducted, as a randomized clinical trial, and the number of cases was small in order to established robust conclusions. Secondly, we did not compare our results in elderly patients to a control group of patients <70 years old with the same ERAS protocol or under the traditional treatment, due to a lack of information in our previous database prior to the start of the study.

## Conclusions

Based on our data from the present multicenter study, ERAS should be implemented without reservations in elderly patients undergoing elective colorectal surgeries, expecting the same goals and benefits as found in other age groups. Barriers in achieving a high compliance with ERAS interventions in elderly patients are common and will require a great effort in the patient education, an intensive plan of pre and postoperative care, and sometimes a change in the surgeons’ management.
